# Supplementary material for: SALpingectomy for STERilisation (SALSTER): study protocol for a Swedish multicentre register-based randomised controlled trial
Source: BMJ Open. 2023 Sep 4;13(9):e071246. doi: 10.1136/bmjopen-2022-071246 (PMC10481827; doi:10.1136/bmjopen-2022-071246)
Supplement: Supplementary data [file bmjopen-2022-071246supp001.pdf]

SALPINGECTOMY AT STERILIZATION

SALSTER

Consent to take part in the SALSTER study involves

- confirming that you have received information about the study and have been given the opportunity to ask questions

- that you agree to participate in the study and that your personal data will be processed as described in the information

- that you are aware that your participation is completely voluntary and that you can cancel your participation without explanation, and without affecting your care and treatment in any future contacts with the health care system

I agree to participate in the SALSTER study

☐ Yes, I do

☐ No

☐ Maybe, but I would like more information

I agree to answer survey questions

☐ Yes

☐ No

2018-09-07

SNAKS Swedish network for national clinical studies in Ob/Gyn

This is the SALSTER Consent form as it appears in a print-out. It has been translated from Swedish with www.DeepL.com/Translator

Women log on to GynOp where they read this text and give consent on-line under the protection of a secured password.
