# Supplementary material for: SALpingectomy for STERilisation (SALSTER): study protocol for a Swedish multicentre register-based randomised controlled trial
Source: BMJ Open. 2023 Sep 4;13(9):e071246. doi: 10.1136/bmjopen-2022-071246 (PMC10481827; doi:10.1136/bmjopen-2022-071246)
Supplement: Author's manuscript [file bmjopen-2022-071246.draft_revisions.pdf]

BMJ Open is committed to open peer review. As part of this commitment we make the peer review history of every article we publish publicly available.

When an article is published we post the peer reviewers' comments and the authors' responses online. We also post the versions of the paper that were used during peer review. These are the versions that the peer review comments apply to.

The versions of the paper that follow are the versions that were submitted during the peer review process. They are not the versions of record or the final published versions. They should not be cited or distributed as the published version of this manuscript.

BMJ Open is an open access journal and the full, final, typeset and author-corrected version of record of the manuscript is available on our site with no access controls, subscription charges or pay-per-view fees (http://bmjopen.bmj.com).

If you have any questions on BMJ Open's open peer review process please email info.bmjopen@bmj.com

BMJ Open

SALpingectomy for STERilisation (SALSTER); Study protocol for a Swedish multicentre register-based randomised controlled trial.

Journal:	*BMJ Open*
Manuscript ID	bmjopen-2022-071246
Article Type:	Protocol
Date Submitted by the Author:	21-Dec-2022

Complete List of Authors:	Magarakis, Leonidas; University of Gothenburg, Department of Obstetrics and Gynecology<br>Idahl, Annika ; Umea Universitet,<br>Sundfeldt, Karin; University of Gothenburg, Department of Obstetrics and Gynecology; Sahlgrenska University Hospital, Department of Obstetrics and Gynecology<br>Liv, Per; Umeå University, Department of Public Health and Clinical Medicine, Section of Sustainable Health<br>Pålsson, Mathias; University of Gothenburg, Department of Obstetrics and Gynecology<br>Strandell, Annika; University of Gothenburg Institute of Clinical Sciences

Keywords:	Minimally invasive surgery < GYNAECOLOGY, Gynaecological oncology < GYNAECOLOGY, GYNAECOLOGY

SCHOLARONE™
Manuscripts

SALpingectomy for STERilisation (SALSTER); Study protocol for a Swedish multicentre register-based randomised controlled trial.

Leonidas Magarakis[a*], Annika Idahl[b], Karin Sundfeldt[a,c], Per Liv[d], Mathias Pålsson[a] and Annika Strandell[a,c]

[a] Department of Obstetrics and Gynecology, Institute of Clinical Sciences, Sahlgrenska Academy, University of Gothenburg, Sweden.

[b] Department of Clinical Sciences, Obstetrics and Gynecology, Umeå University, Umeå, Sweden.

[c] Department of Obstetrics and Gynecology, Sahlgrenska University Hospital, Gothenburg, Sweden.

[d] Department of Public Health and Clinical Medicine, Umeå University, Umeå, Sweden.

*Corresponding author:

Leonidas Magarakis, Department of Obstetrics and Gynecology, Skåne University Hospital, Jan Waldenströms gata 47, SUS, Malmö, Sweden.

email: leonidas.magarakis@gu.se, tel: +4640332541

Word count abstract: 250 (Limit 250)

Word count manus: 3944 (Limit 4000)

Keywords: Sterilisation, Salpingectomy, Tubal ligation, Randomised controlled trial, Ovarian cancer

For peer review only

Abstract

Introduction

Salpingectomy is currently suggested as an alternative to tubal ligation for sterilisation. Precursor lesions of ovarian carcinoma can be found in the Fallopian tubes; thus, salpingectomy could possibly reduce the incidence. Most of the existing trials on safety are small, on caesarean section and report on surrogate ovarian function measures. Randomised trials in laparoscopy are lacking. Well-designed trials are needed to evaluate safety of laparoscopic opportunistic salpingectomy.

Methods and analysis

In SALSTER, a national register-based randomised controlled non-inferiority trial, women <50 years wishing laparoscopic sterilisation will be randomised to either salpingectomy or tubal ligation. The Swedish National Quality Register of Gynecological Surgery (GynOp) will be used for inclusion, randomisation, and follow-up. Primary outcomes are any complications up to eight weeks postoperatively, and age at menopause. Both outcomes are measured with questionnaires, complications are also assessed by a gynaecologist. In a nested trial, ovarian function will be evaluated comparing the mean difference of anti-Müllerian hormone, assessed preoperatively and one year after surgery.

Ethics and dissemination

Performing salpingectomy for sterilisation has become increasingly common, despite the unclear risk-benefit balance. SALSTER studies the safety of salpingectomy compared with tubal ligation. Regardless of the result, SALSTER will provide gynaecologists with high quality evidence to inform women to decide on salpingectomy or not. The central ethical

For peer review only - http://bmjopen.bmj.com/site/about/guidelines.xhtml

review board of Gothenburg, Sweden (Dnr. 316-18) approved the trial in June 2018. Results will be presented at scientific congresses and published in peer reviewed scientific journals. The results will be communicated through professional organisations and research networks.

Registration details

ClinicalTrials.gov, NCT03860805. Registered March 4th, 2019. Study protocol last updated November 21st, 2021.

Strengths and limitations of the study

- The register-based randomised controlled trial combines the advantages of two study designs: the randomised trial with unbiased allocation to minimise confounding and the observational register study with an automated and cost-efficient follow-up.
- Using the GynOp register as a platform allows all trial components (identification of eligible patients, communication regarding study information and giving informed consent, randomisation, and follow-up questionnaires) to be conducted within the register.
- The use of the Swedish personal identification number allows cross-linking of the study cohort with multiple registers for the long-term follow-up.
- The multicentre design enhances the generalisability of the results.
- The nature of the trial makes blinding of the patients very difficult and impossible for the surgeons.

INTRODUCTION

The use of salpingectomy as a sterilisation procedure is increasing, due to the theory of high-grade serous ovarian carcinoma (HGSC) originating from the Fallopian tube. Epithelial

For peer review only - http://bmjopen.bmj.com/site/about/guidelines.xhtml

ovarian cancer (EOC) is a group of heterogeneous malignancies regarding origin, molecular biology, morphology, gene expression, and clinical behaviour. Precancerous lesions, serous tubal intraepithelial carcinomas (STIC), detected in the tubal epithelium are suggested to be the origin of EOC, particularly HGSC. Dysplastic cells may shed from STIC lesions and implant on the ovaries and/or peritoneum and develop into HGSC.[1] Opportunistic salpingectomy to remove the potential site of origin as a preventive measure is therefore suggested for women who wish permanent sterilisation.[2 3]

Tubal ligation is by itself associated with some protection against EOC.[4] Fallopian tubes may act as a conduit of either malignant or normal cells from the endometrial cavity to the ovaries. These cells may give rise to endometrioid and clear-cell carcinomas directly or indirectly by malignant transformation of benign conditions such as endometriosis.[5] Possibly, salpingectomy could add to the protective effect of tubal ligation by removing the fimbriated end of the Fallopian tubes where STIC lesions may develop.[4 6 7]

Several gynaecological societies recommend physicians to inform women planned to undergo sterilisation, that bilateral salpingectomy instead of tubal ligation, is an option.[2 3] This recommendation is based on observational studies showing that *indicated* salpingectomy compared with no surgery, is associated with a decreased EOC incidence.[4 6 7] The effect size of *opportunistic* salpingectomy compared with tubal ligation is unknown.

There are safety concerns, since salpingectomy increases surgical trauma compared with tubal ligation. This may increase perioperative complications and may also affect blood and nerve supply to the ovaries, impairing ovarian function, and possibly, in the long term, cause an earlier menopause.[8] Systematic reviews comparing salpingectomy with tubal ligation for

For peer review only - http://bmjopen.bmj.com/site/about/guidelines.xhtml

safety outcomes such as reoperation, intraoperative complications, blood loss, wound infections etc, have identified studies with various limitations.[9] All published randomised controlled trials (RCTs) are small and conducted at caesarean section. They report on surrogate measures of endocrine function and demonstrate no difference in the short term.[10-12] Many of the published cohort studies are small and underpowered to study complications. Sterilisation is more commonly performed by laparoscopy, especially after the hysteroscopic salpingeal occluding technique with permanent implants was withdrawn from the market due to adverse effects.[13] No trial has reported on the outcome EOC. A large retrospective cohort study detected no difference in time to menopausal symptoms when comparing women who had undergone salpingectomy or tubal ligation. However, the follow-up period was insufficiently short to analyse menopausal symptoms.[14] Well-designed randomised trials of laparoscopic sterilisation procedures are needed to compare salpingectomy with tubal ligation regarding both surgical outcomes and clinical endpoints of ovarian function.

This register-based randomised trial will study the safety of laparoscopic salpingectomy for sterilisation compared with tubal ligation. The specific aim is to analyse if the risk of complications and hormonal side effects do not increase beyond pre-defined non-inferiority margins after salpingectomy compared with tubal ligation.

METHODS AND ANALYSIS

General study design

SALSTER, a national register-based, randomised controlled trial (R-RCT) will compare two laparoscopic procedures for sterilisation: salpingectomy and tubal ligation. In the long term, the EOC outcome will be pooled with data from the Hysterectomy and OPPortunistic

For peer review only - http://bmjopen.bmj.com/site/about/guidelines.xhtml

SAlpingectomy (HOPPSA) trial in an independent patient data (IPD) meta-analysis. The aim is to demonstrate that opportunistic salpingectomy is superior to leaving the tubes *in situ* at sterilisation or hysterectomy, regarding risk reduction of EOC.[15]

In the primary analyses, SALSTER will test the hypotheses that salpingectomy compared with tubal ligation for laparoscopic sterilisation,

- does not increase the risk for complications perioperatively and up to eight weeks postoperatively.
- does not cause earlier menopause, assessed as age at onset of natural menopause.

The GynOp register

The SALSTER trial is conducted within the Swedish National Quality Register of Gynecological Surgery (GynOp).[16] GynOp is used by all gynaecological departments in Sweden. Inclusion and participation in national quality registers in Sweden is regulated by law[17]; patients are informed of their inclusion in the register, with an "opt-out" clause which, if activated, enables the patient to have all his or her data removed from the register. The GynOp database is approved for use by health-care systems under the supervision of the Swedish Data Protection Authority. All information is stored on secured servers at Region Västerbotten. Background health data, information on surgical procedures, diagnoses, complications at eight weeks and one year postoperatively are routinely recorded in GynOp. Women planned for gynaecologic surgery receive a personal password that allows them to logon to GynOp to answer pre-operative and follow-up questionnaires. Data input in GynOp is mainly web-based, but printouts of questionnaires can be used if needed. The data collection forms and questionnaires are available from www.gynop.org on request.

For peer review only - http://bmjopen.bmj.com/site/about/guidelines.xhtml

All gynaecological departments reporting data to the register received information about the trial and were automatically included unless a department actively declined participation. A list of gynaecological departments participating in the study can be provided by the GynOp office in Umeå on demand. Both regional and academic gynaecological departments are participating in the study. The Swedish network for National Clinical Studies in Obstetrics and Gynecology (SNAKS) is actively involved and improves collaboration between health care providers engaged in the trial.[18]

A specific SALSTER application has been added to GynOp to complement existing routines. This module includes screening of eligibility, presentation of study information and opportunity to give informed consent on-line, as well as randomisation and trial-specific questionnaires pre-operatively and for follow-up.

Preoperatively, basic baseline demographic variables are registered routinely. Added to these variables are questions on menstruation pattern, age at menarche, duration of breast feeding, previous and present use of hormonal contraceptives and previous Chlamydia infection or salpingitis to assess factors suggested to effect risk for EOC. Furthermore, the Menopause Rating Scale (MRS)[19] was added.

MRS is a validated questionnaire available in several languages, including Swedish. It has 11 questions on sweating, heart discomfort, sleep problems, depressive mood, irritability, anxiety, physical and mental exhaustion, sexual problems, bladder problems, vaginal dryness, and joint and muscular function, to which patients respond in a five-grade Likert scale.[20]

Perioperative variables in GynOp are type of anaesthesia, any pathological finding in the abdomen, procedure(s) performed, complications, use of antibiotics, operative time, route of specimen removal from the abdomen, blood loss, type of suturing and codes for surgery.

For peer review only

SALSTER-specific questions concern total number and size of trocars used, method for tubal ligation, type of devices applied for salpingectomy and tubal ligation, specific questions on method of specimen extraction and need to suture the muscle fasciae following specimen evacuation.

GynOp automatically sends questionnaires to the patients electronically at eight weeks and one year postoperatively, to assess use of analgesics, bleeding, low urinary tract symptoms, sick leave, time to daily activities, satisfaction after surgery, complications and their treatment. If no answer is received, two reminders are sent automatically. Patient-reported complications are assessed and documented by a gynaecologist. Any complication is registered according to the Clavien-Dindo classification.[21] No amendments have been made to the eight-weeks questionnaire.

The one-year questionnaire holds questions relating to pain experience, oestrogen treatment, symptoms from vagina, bladder and rectum, sexual intercourse last three months, coitus pain, result and satisfaction after surgery, complications, treatment of complications, hospital care, and sick leave. The one-year follow-up questionnaire has been supplemented with trial-specific questions on oestrogen and/or progesterone hormonal treatments and their indication, MRS, menstruation pattern, unintended pregnancies and their outcomes, and smoking habits. If complications are reported, these are assessed by a gynaecologist. Two routine reminders are sent.

Routinely there is no further follow up from GynOp. For trial participants questionnaires are sent every other year until the age of 55. Questions relate to the use of menopausal hormone therapy (MHT) or oestrogen and/or progesterone hormonal treatments and their indication, MRS, bleeding pattern, smoking habits, and unintended pregnancies and their outcomes.

For peer review only - http://bmjopen.bmj.com/site/about/guidelines.xhtml

Eligibility

All patients planned for laparoscopic sterilisation are automatically screened for eligibility in the trial by the GynOp software. Potential trial participants can read on-line the SALSTER information and answer the specific study questions. Paper printouts are also available in which case a medical administrator registers the information in GynOp by using a login with a two-factor authentication system. Patients may also be informed about the trial at an out-patient clinic visit when the decision on sterilisation is taken. Informed consent can be given either on-line within GynOp or by signing a paper document at any time point before randomisation. The consent is kept safe according to established research routines. Inclusion and exclusion criteria are summarised in Table 1.

Table 1. Eligibility criteria for women participating in SALSTER

Inclusion criteria	*Exclusion criteria*
Scheduled for laparoscopic sterilisation.	50 years or older.<br>Not able to understand oral and written study information.<br>Previously treated with either chemo-, radio- or hormonal therapy which may negatively affect ovarian function.

Randomisation and blinding

The randomisation module in GynOp randomly allocates women 1:1 to either salpingectomy or tubal ligation, stratified by age and centre. Timing of randomisation is as close as possible to the time of surgery. The randomisation is performed on-line by the examining/operating gynaecologist or assistant with an immediate allocation response.

The nature of the trial makes blinding of patients very difficult and impossible for surgeons. Our intention is to avoid revealing information about which type of surgery was performed and we ask trial participants not to read their on-line medical records. Blinding of patients is further aggravated as a detailed preoperative information is given including the number of

For peer review only - http://bmjopen.bmj.com/site/about/guidelines.xhtml

scars associated with each procedure. In general, tubal ligation requires only one accessory port whereas salpingectomy requires at least two.

Interventions

Both interventions are planned as laparoscopic procedures. If the allocated procedure cannot be executed because of either unexpected pathology or high risk for serious intraoperative complications, the surgical procedure that was eventually done will be registered in GynOp, but the individual still contributes with follow-up data. The same applies if extra surgical procedures are needed or in case of conversion to laparotomy where all surgical interventions are registered.

Follow-up

Hospital staff routinely register data in GynOp at the end of every surgical procedure and at discharge. In case of a complication the surgeon registers the event. Responsible surgeon assesses the eight-weeks and one-year questionnaires and in suspicion of a complication or unsatisfactory surgical results, a consultation is arranged. Any adverse effect is registered in GynOp. If there is no response after two routine reminders a member of the steering group contacts the department. In every department, a responsible physician will check responses and completeness of questionnaires at different time points. In case of an adverse event, any need for medical treatment to trial participants is covered by the Swedish health care system according to the Swedish law.

Outcomes

For peer review only - http://bmjopen.bmj.com/site/about/guidelines.xhtml

The trial has two primary outcomes, one in the short- and one in the long-term. Secondary outcomes are registered in the short-, intermediate- and long-term. The primary and secondary outcomes are listed in Table 2.

Table 2. Outcomes in SALSTER

Time interval	***Primary outcomes***	***Secondary outcomes***
Short term (up to 8 weeks)	Any complication	Severe complications<br>Operative time<br>Perioperative blood loss.<br>Length of hospital stay
Intermediate term (one year after surgery)		Complications according to Clavien-Dindo<br>Complications according to the existing questions on complications in GynOp<br>Subsequent surgery on uterus, salpinges and/or ovaries<br>Pregnancy rate
Long term	Age at onset of natural menopause	Age at the start of the perimenopausal state<br>Length of the perimenopausal state<br>Change in menopausal symptom score<br>Use of menopausal hormone therapy at any time during follow-up<br>Subsequent surgery on uterus, salpinges and/or ovaries<br>Pregnancy rate<br>Secondary expressions of oestrogen deficiency<br>Epithelial ovarian cancer

Any complication up to eight weeks post-operatively, is retrieved directly from the GynOp database. The outcome includes any complication occurring per-operatively, diagnosed at postoperative emergency visits, or noted by the patient and assessed by the physician in the eight-weeks questionnaire. The complication is further categorised as mild or severe, by organ damaged, and is graded according to the Clavien-Dindo classification. These categorised variables will be analysed as secondary outcomes.

Age at onset of natural menopause, defined as twelve months of amenorrhea, is assessed by analysing reported bleeding pattern in the study-specific questionaries sent every other year.

For peer review only - http://bmjopen.bmj.com/site/about/guidelines.xhtml

Women with MHT prescription, oestrogen and/or progesterone hormonal treatments, or a subsequent hysterectomy will not be included in this primary outcome, since they do not have a natural menopause.

The secondary short-term outcomes relate to the surgery and the in-hospital care as registered in GynOp. Secondary intermediate-term outcomes are retrieved from GynOp and other national quality and health registers. Secondary long-term outcomes such as length of and age at the start of perimenopausal state will be assessed by the trial-specific questionnaires describing bleeding pattern. Need for MHT will be assessed by every-other-year questionaries and through The Drug Prescription Register up to 30 years after surgery. Uterine and adnexal surgery that occurs after the primary surgery will be assessed through GynOp at one year and The Patient register lifelong after surgery. Unintended pregnancies and their outcomes will be registered through the trial-specific questionnaires. If intermediate term outcomes on ovarian function show a difference between groups, consequences of oestrogen deficiency, i.e., fractures related to osteoporosis and cardio-vascular events will be assessed through The Patient register. In the long-term, data from SALSTER will be pooled with data from the ongoing HOPPSA trial to analyse the incidence of epithelial ovarian cancer. Data will be retrieved through The Swedish Cancer Register, The Swedish Quality Register for Gynaecological Cancer, The Swedish Cause of Death Register and The Swedish Population Register and at lifelong follow-up.

Data monitoring and data management

Each surgical procedure in GynOp automatically receives a unique identification code number. This number is used in the trial to assign individual data, thus protecting confidentiality. The number of individuals randomised in the trial is continuously monitored

For peer review only - http://bmjopen.bmj.com/site/about/guidelines.xhtml

by the GynOp's administrators. Numbers of recruited and percentage of eligible women per participating clinic are reported every three months on the GynOp website and through the SNAKS network which enhances communication between the research group and the departments participating in the trial. Regular online meetings are being held updating departments on the progress of the trial, and information is shared on recruiting performance. An independent appointed Data Safety Monitoring Board has performed an interim analysis when 50% of the target sample size was reached, according to the original plan, and gave clearance for the study to continue recruiting patients.

Patient and public involvement

Women in reproductive age in the general population were involved at an early phase of the planning, regarding choice of outcomes and development of the written study information. A short explanation of the research question and the intended study protocol in lay language with suggested outcomes were distributed among volunteers in waiting rooms at gynaecology departments in Sweden. Open and specific questions were asked concerning the relevance of the trial, the design, the outcomes, any missing issues, or missing outcomes. Questions associated with the draft of the written study information related to readability, unnecessary or missing information. Women were also asked to rate the importance of receiving information about potential risks associated with opportunistic salpingectomy.

STATISTICS

Sample size calculations

Primary short-term outcome: *any complication* up to eight weeks

For peer review only - http://bmjopen.bmj.com/site/about/guidelines.xhtml

Complications to laparoscopic tubal ligation was registered in GynOp at a rate of 13.6% from 2010 to 2017. An increase of 3% is estimated after salpingectomy. If the non-inferiority margin is defined as +10%, the upper limit of the two-sided 95% CI (α=0.05) for the difference between the salpingectomy and the tubal ligation groups shall not be above the +10% with a probability of 80% (β=20%). To demonstrate non-inferiority, 411 women per randomisation group are needed (based on a two-sided Farrington-Manning test).[22] For protection against a 10% loss to follow-up, the target sample was determined at 914. The interim analysis revealed that 5% of randomised women interrupted their participation. For protection against this loss, the target sample size was increased to 968.

Primary long-term outcome: *age at onset of menopause*

Age at menopause on a Swedish population level was reported to be in mean 51.5 years and SD was estimated at 3.0. A decrease of one year is estimated after salpingectomy. If the non-inferiority margin is defined as two years, the upper limit of the two-sided 95% CI (α=0.05) for the difference between the salpingectomy group and the tubal ligation group shall not be above two years with a probability of 80% (β=20%). To demonstrate non-inferiority, 143 women per randomisation group are needed (two-sided non-parametric permutation test for comparison of two means). Considering exclusion of women without a natural menopause (30%), 5% of randomised women interrupting participation before the eight-weeks questionnaire, and 15% loss during the 20 years long follow-up, approximately 572 women are needed for recruitment.

Statistical plan

Both “intention to treat”, and “per protocol” analyses will be performed. For non-inferiority design, the “per protocol” analysis will be the primary.

For peer review only - http://bmjopen.bmj.com/site/about/guidelines.xhtml

Any complication will be presented as numbers along with percentages with 95% CI and the *age at onset of menopause* will be presented as mean and standard deviations, as well as with median and quartiles. The two primary analyses measure different outcomes at different time points and will be published in separate articles. As they also test two different hypotheses, we will refrain from adjusting the 5% significance level for multiplicity.

Analyses of the short-term outcome *any complication*

Primary analysis: To account for the lack of independence introduced by the stratification of the randomisation, we will estimate the difference in the complication risk between the two randomised groups with a 95% CI using a generalised estimation equation (GEE) with logistic link function, marginalised over centre, and adjusted for age. The 95% CI of the risk difference will be estimated from the GEE-model using the delta method. The upper limit of the 95% CI shall not exceed the non-inferiority margin of 10%. As a sensitivity analysis, the unadjusted 95% CI for the difference in complications will be calculated according to Ferrington-Manning.[22] Furthermore, unadjusted risk ratio (RR) and adjusted RR with 95% CI will also be calculated in secondary analyses using a GEE Poisson model with robust standard errors.

Analyses of the long-term outcome *age at menopause*

The primary analysis will be a mixed effect model with adjustment for age as fixed effect and centre as random effect, from which a two-sided 95% CI for the mean difference will be constructed. The upper limit of the 95% CI shall not exceed the non-inferiority margin of two years for non-inferiority to be established. A sensitivity analysis without adjustment will be

For peer review only

conducted by constructing a 95% CI for the mean difference using Fisher's non-parametric permutation test.

Missing data on the primary outcomes will be replaced with multiple imputation using fully conditional specification in the main analysis. In addition, a complete case analysis will be conducted. If both analyses of the two primary outcomes demonstrate non-inferiority, a common conclusion on the safety of the intervention can be inferred. However, the long period between these analyses will entail separate conclusions on complications and age at menopause, in a temporal order.

For other unadjusted comparisons between the two randomised groups Fisher's non-parametric permutation test will be used for continuous variables, Mantel-Haenszel Chi^2-test for ordered categorical variables, Fisher's exact test for dichotomous variables and Chi^2-test for non-ordered categorical variables. For dichotomous outcomes, a two-sided 95% CI for the difference in proportions between groups will be calculated as well as risk ratios with 95% CI. For continuous outcomes, two-sided 95% CIs for the difference in means between groups will be calculated. Also, adjusted analyses will be conducted.

All results from the secondary analysis will be given with estimates, 95% CI and two-sided p-values, as well as unadjusted and adjusted RR with 95% CI. The analyses of the secondary endpoints will be mainly explanatory.

A detailed statical analysis plan (SAP) will be written before data retrieval and published at the trial's site at ClinicalTrials.gov. Updates and changes in the planned statistical analyses will be published there.

For peer review only - http://bmjopen.bmj.com/site/about/guidelines.xhtml

NESTED TRIAL OF ANTI-MÜLLERIAN HORMONE LEVELS

A biochemical measure of ovarian function is the serum level of AMH, a product of granulosa cells of the preantral and small antral follicles in the ovaries.[23] There is a theoretical rationale that salpingectomy may disturb the vascular and nervous supply to the ovary, or disrupt paracrine signalling, possibly causing impairment in ovarian function.[8] In the main trial, the primary outcome for ovarian function is based on clinical symptoms related to menopause. To strengthen the hypothesis of non-inferiority for ovarian function if salpingectomy is performed, an analysis of AMH is planned in a subset of patients.

Consecutive patients in SALSTER are asked for blood samples. Specific written and oral information is provided, and informed consent is signed. Blood samples are drawn at baseline and after one year. Seven hospitals are engaged in this nested trial. Samples are handled according to laboratory instructions, centrifugated, frozen within two days and stored in a biobank for later analysis, when the entire cohort will be analysed at the same time.

Results will be available after one year of follow-up and added manually to the GynOp dataset. Patients wishing to be informed about their AMH levels result will be contacted. AMH levels will be compared between the salpingectomy vs. tubal ligation groups and presented both in absolute and relative measures. Primary endpoint is absolute change in AMH from baseline to one year after surgery.

If non-inferiority is defined as 0.2 mg/L AMH, the upper limit of the two-sided 95% CI for the difference in change between the two groups shall not exceed 0.2 (SD for change 0.45)

For peer review only - http://bmjopen.bmj.com/site/about/guidelines.xhtml

with a probability of 80% (β=20%), and an estimation of up to 0.0 larger change (no difference in change) in the salpingectomy group, 81 patients per randomisation group is needed to show non-inferiority. Estimating a 20% loss to follow-up (a second blood sample not taken), 204 patients will be recruited in this nested trial. A two-sided 95% CI for the mean difference in absolute change in AMH will be constructed using a mixed effect model with adjustment for age as fixed effect and centre as random effect. Fisher's non-parametric permutation test will be applied for the unadjusted analysis.

ETHICS AND DISSEMINATION

Even though EOC is not the most common gynaecological cancer it carries the worst prognosis due to early spread and vague symptomatology, making diagnosis difficult at an early stage. Based on the theory that the most common and aggressive form, HGSC may arise from the epithelium of the Fallopian tubes, the practice of opportunistic salpingectomy has rapidly gained popularity. Well-designed trials have not been performed to study the safety profile of salpingectomy compared with tubal ligation regarding complications and the effect on ovarian function. SALSTER will assess if salpingectomy is as safe as tubal ligation. The withdrawal of hysteroscopic sterilisation made the trial ethically reasonable to design since the less invasive hysteroscopic procedure for sterilisation was not available anymore.[13] Regardless of the result, the trial will provide gynaecologists with high quality evidence to inform women, who can decide on having their tubes removed or not. If no additional risk is found, salpingectomy can be a recommended option. If not, the risks and benefits should be considered when counselling women wishing permanent surgical sterilisation.

For peer review only - http://bmjopen.bmj.com/site/about/guidelines.xhtml

SALSTER does not have EOC as a primary outcome for several reasons: There is a parallel trial, HOPPSA, which has EOC as a long-term primary outcome. At inclusion, the patients in HOPPSA are older than those in SALSTER, which implies a shorter time-to-event than in SALSTER. Also, hysterectomy is a more frequent procedure than sterilisation in Sweden, implying faster recruitment to the target sample size. Thus, the HOPPSA trial is more suited to investigate and conclude on EOC as a primary outcome. Furthermore, the plan for SALSTER is to contribute data to be pooled with HOPPSA data for the evaluation of the effect of opportunistic salpingectomy on EOC. A combined SAP will be written for an IPD meta-analysis combining HOPPSA and SALSTER.

The results of this trial will be presented at national as well as international scientific congresses and several publications are planned in international scientific journals. All results will be presented on aggregated level, without any possibility to identify individuals. SNAKS will help to spread the results of this trial to its network of gynaecological departments in Sweden. Updates of results will be presented at the annual meetings of the Swedish Society of Obstetrics and Gynecology.

The SALSTER trial was approved by the central ethical review board in Gothenburg, Sweden June 18th, 2018 (Dnr. 316-18). The first patient was randomised April 4th, 2019. The trial is recruiting, and 864 women had been randomised August 31st, 2022.

Authors' contribution

AS initiated the trial, designed, and drafted the first study protocol. AI engaged in the revision and editing of the protocol. AI and MP are the primary contact persons with the GynOp register. KS contributes with ovarian tumour biology experience. LM initiated the AMH nested trial. AS, AI, KS, MP, and LM approved the study protocol. AS applied to the

For peer review only - http://bmjopen.bmj.com/site/about/guidelines.xhtml

Swedish Ethical Review Authority. PL wrote the statistical plan. LM wrote the first draft of this manuscript which was revised by AS, AI, KS, and MP. All committed authors approved the final version of this manuscript.

Principal investigator: Annika Strandell, contact email: annika.strandell@vgregion.se

Funding statement

This work was supported by the Swedish Cancer Society (21 1408 Pj), the Lena Wäppling foundation, the Swedish state under the agreement between the Swedish government and the county councils, the ALF-agreement (ALFGBG-965130 and ALF RegVB-969584), Umeå University and Center for clinical research, county of Värmland grant number (LIVFOU-929703). The funders were not involved in study design; collection, management, analysis, and interpretation of data; writing of the report; and the decision to submit the report for publication.

Competing interest statement

None of the other authors have any conflicts of interest.

REFERENCES

1. Prat J. New insights into ovarian cancer pathology. *Annals of oncology : official journal of the European Society for Medical Oncology* 2012;23 Suppl 10:111-7. doi: https://doi.org/10.1093/annonc/mds300
2. Salvador S, Scott S, Francis JA, et al. No. 344-Opportunistic Salpingectomy and Other Methods of Risk Reduction for Ovarian/Fallopian Tube/Peritoneal Cancer in the General Population. *Journal of obstetrics and gynaecology Canada : JOGC* 2017;39(6) doi: https://10.1016/j.jogc.2016.12.005
3. ACOG Committee Opinion No. 774: Opportunistic Salpingectomy as a Strategy for Epithelial Ovarian Cancer Prevention. *Obstetrics and gynecology* 2019;133(4) doi: https://10.1097/AOG.0000000000003164

For peer review only - http://bmjopen.bmj.com/site/about/guidelines.xhtml

4. Madsen C, Baandrup L, Dehlendorff C, et al. Tubal ligation and salpingectomy and the risk of epithelial ovarian cancer and borderline ovarian tumors: a nationwide case-control study. *Acta obstetricia et gynecologica Scandinavica* 2015;94(1):86-94. doi: https://doi.org/10.1111/aogs.12516
5. Cochrane DR, Tessier-Cloutier B, Lawrence KM, et al. Clear cell and endometrioid carcinomas: are their differences attributable to distinct cells of origin? *The Journal of pathology* 2017;243(1) doi: https://10.1002/path.4934
6. Lessard-Anderson CR, Handlogten KS, Molitor RJ, et al. Effect of tubal sterilization technique on risk of serous epithelial ovarian and primary peritoneal carcinoma. *Gynecologic oncology* 2014;135(3) doi: https://doi.org/10.1016/j.ygyno.2014.10.005
7. Falconer H, Yin L, Grönberg H, et al. Ovarian cancer risk after salpingectomy: a nationwide population-based study. *Journal of the National Cancer Institute* 2015;107(2):410. doi: https://doi.org/10.1093/jnci/dju410
8. Kotlyar A, Gingold J, Shue S, et al. The Effect of Salpingectomy on Ovarian Function. *Journal of minimally invasive gynecology* 2017;24(4) doi: https://10.1016/j.jmig.2017.02.014
9. Magarakis L, Idahl A, Särnqvist C, et al. Efficacy and safety of sterilisation procedures to reduce the risk of epithelial ovarian cancer: a systematic review comparing salpingectomy with tubal ligation. *The European journal of contraception & reproductive health care : the official journal of the European Society of Contraception* 2021 doi: https://10.1080/13625187.2021.2010041
10. Ganer Herman H, Gluck O, Keidar R, et al. Ovarian reserve following cesarean section with salpingectomy vs tubal ligation: a randomized trial. *American journal of obstetrics and gynecology* 2017;217(4):472.e1-72.e6. doi: https://doi.org/10.1016/j.ajog.2017.04.028
11. Garcia C, Moskowitz OM, Chisholm CA, et al. Salpingectomy Compared With Tubal Ligation at Cesarean Delivery: A Randomized Controlled Trial. *Obstetrics and gynecology* 2018;132(1):29-34. doi: https://doi.org/10.1097/AOG.0000000000002674
12. Subramaniam A, Blanchard CT, Erickson BK, et al. Feasibility of Complete Salpingectomy Compared With Standard Postpartum Tubal Ligation at Cesarean Delivery: A Randomized Controlled Trial. *Obstetrics and gynecology* 2018;132(1):20-7. doi: https://doi.org/10.1097/AOG.0000000000002646
13. Problems Reported with Essure. Available from: https://www.fda.gov/medical-devices/essure-permanent-birth-control/problems-reported-essure [Accessed 11th November 2022].
14. Hanley GE, Kwon JS, McAlpine JN, et al. Examining indicators of early menopause following opportunistic salpingectomy: a cohort study from British Columbia, Canada. *American journal of obstetrics and gynecology* 2020;223(2):221.e21-21.e11. doi: https://doi.org/10.1016/j.ajog.2020.02.005
15. Idahl A, Darelius A, Sundfeldt K, et al. Hysterectomy and opportunistic salpingectomy (HOPPSA): study protocol for a register-based randomized controlled trial. *Trials* 2019;20(1) doi: https://10.1186/s13063-018-3083-8
16. The Swedish National Quality Register of Gynecological Surgery. Available from: http://www2.gynop.se/home/ [Accessed 7th November 2022].
17. Swedish National Quality Registries. Available from: https://www.kvalitetsregister.se/en/kvalitetsregister/omnationellakvalitetsregister.52218.html [Accessed 7 November 2022].
18. Swedish network for national clinical studies in Obstetrics and Gynecology. Available from: https://www.snaks.se [Accessed 7th November 2022].

For peer review only - http://bmjopen.bmj.com/site/about/guidelines.xhtml

19. Heinemann K, Ruebig A, Potthoff P, et al. The Menopause Rating Scale (MRS) scale: a methodological review. *Health and quality of life outcomes* 2004;2 doi: https://10.1186/1477-7525-2-45
20. Menopause Rating Scale. Available from: http://www.menopause-rating-scale.info/documents/MRS_English.pdf [Accessed 7th November 2022].
21. Dindo D, Demartines N, Clavien PA. Classification of surgical complications: a new proposal with evaluation in a cohort of 6336 patients and results of a survey. *Annals of surgery* 2004;240(2) doi: https://10.1097/01.sla.0000133083.54934.ae
22. Farrington CP, Manning G. Test statistics and sample size formulae for comparative binomial trials with null hypothesis of non-zero risk difference or non-unity relative risk. *Statistics in medicine* 1990;9(12) doi: https://10.1002/sim.4780091208
23. Vigier B, Picard JY, Tran D, et al. Production of anti-Müllerian hormone: another homology between Sertoli and granulosa cells. *Endocrinology* 1984;114(4) doi: https://10.1210/endo-114-4-1315

For peer review only

SPIRIT schedule of enrolment, interventions, and assessments in the SALSTER trial according to the SPIRIT guidelines

			STUDY PERIOD					
	**Enrolment**	**Allocation**	**Post-allocation**					
**TIMEPOINT****	***0-30 days before allocation***	**0**	***Peri-operative***	***At discharge***	***8 weeks***	***1 year***	***Every other year up to 55 years of age***	***20-30 years***
**ENROLMENT:**								
**Eligibility screen**	X							
**Informed consent**	X							
Health questionnaires	X							
Factors that may affect the risk for EOC	X							
MRS	X							
**Allocation**		X						
**INTERVENTIONS:**								
Bilateral salpingectomy			X					
Tubal ligation			X					
**ASSESSMENTS:**								
Baseline characteristics	X	X						
Perioperative variables			X	X				
SALSTER specific operative variables			X	X				
Severe complications			X	X	X	X		
Complications			X	X	X	X		
Age at the start of perimenopausal state							X	
Length of perimenopausal state							X	
Change in menopausal symptom score						X	X	
Use of MHT						X	X	
***Subsequent* surgery on uterus, salpinges and/or ovaries**						X	X	
Pregnancy						X	X	
EOC								X
**Secondary expressions of estrogen deficiency**								X

Abbreviations: EOC= Epithelial Ovarian Cancer, MRS= Menopause rating scale, MRT = Menopausal Hormone Therapy, PBL= Perioperative Blood Loss, SALSTER= SALpingectomy for STERilisation.

For peer review only

SPIRIT

STANDARD PROTOCOL ITEMS: RECOMMENDATIONS FOR INTERVENTIONAL TRIALS

SPIRIT 2013 Checklist: Recommended items to address in a clinical trial protocol and related documents*

Section/item	Item No	Description	Addressed on page number
**Administrative information**			
Title	1	Descriptive title identifying the study design, population, interventions, and, if applicable, trial acronym	1
Trial registration	2a	Trial identifier and registry name. If not yet registered, name of intended registry	3
	2b	All items from the World Health Organization Trial Registration Data Set	-
Protocol version	3	Date and version identifier	3
Funding	4	Sources and types of financial, material, and other support	21
Roles and responsibilities	5a	Names, affiliations, and roles of protocol contributors	1, 20
	5b	Name and contact information for the trial sponsor	20
	5c	Role of study sponsor and funders, if any, in study design; collection, management, analysis, and interpretation of data; writing of the report; and the decision to submit the report for publication, including whether they will have ultimate authority over any of these activities	21
	5d	Composition, roles, and responsibilities of the coordinating centre, steering committee, endpoint adjudication committee, data management team, and other individuals or groups overseeing the trial, if applicable (see Item 21a for data monitoring committee)	-
**Introduction**			
Background and rationale	6a	Description of research question and justification for undertaking the trial, including summary of relevant studies (published and unpublished) examining benefits and harms for each intervention	3 - 5
	6b	Explanation for choice of comparators	3 - 5
Objectives	7	Specific objectives or hypotheses	5
Trial design	8	Description of trial design including type of trial (eg, parallel group, crossover, factorial, single group), allocation ratio, and framework (eg, superiority, equivalence, noninferiority, exploratory)	5

For peer review only

Methods: Participants, interventions, and outcomes

Study setting	9	Description of study settings (eg, community clinic, academic hospital) and list of countries where data will be collected. Reference to where list of study sites can be obtained	5 - 6
Eligibility criteria	10	Inclusion and exclusion criteria for participants. If applicable, eligibility criteria for study centres and individuals who will perform the interventions (eg, surgeons, psychotherapists)	9, Table 1
Interventions	11a	Interventions for each group with sufficient detail to allow replication, including how and when they will be administered	9 - 10
	11b	Criteria for discontinuing or modifying allocated interventions for a given trial participant (eg, drug dose change in response to harms, participant request, or improving/worsening disease)	10
	11c	Strategies to improve adherence to intervention protocols, and any procedures for monitoring adherence (eg, drug tablet return, laboratory tests)	6 - 8
	11d	Relevant concomitant care and interventions that are permitted or prohibited during the trial	9
Outcomes	12	Primary, secondary, and other outcomes, including the specific measurement variable (eg, systolic blood pressure), analysis metric (eg, change from baseline, final value, time to event), method of aggregation (eg, median, proportion), and time point for each outcome. Explanation of the clinical relevance of chosen efficacy and harm outcomes is strongly recommended	10 - 11, Table 2
Participant timeline	13	Time schedule of enrolment, interventions (including any run-ins and washouts), assessments, and visits for participants. A schematic diagram is highly recommended (see Figure)	10 - 11, Figure 1
Sample size	14	Estimated number of participants needed to achieve study objectives and how it was determined, including clinical and statistical assumptions supporting any sample size calculations	13-17
Recruitment	15	Strategies for achieving adequate participant enrolment to reach target sample size	6-9

Methods: Assignment of interventions (for controlled trials)

Allocation:

Sequence generation	16a	Method of generating the allocation sequence (eg, computer-generated random numbers), and list of any factors for stratification. To reduce predictability of a random sequence, details of any planned restriction (eg, blocking) should be provided in a separate document that is unavailable to those who enrol participants or assign interventions	9

Allocation concealment mechanism	16b	Mechanism of implementing the allocation sequence (eg, central telephone; sequentially numbered, opaque, sealed envelopes), describing any steps to conceal the sequence until interventions are assigned	9
Implementation	16c	Who will generate the allocation sequence, who will enrol participants, and who will assign participants to interventions	9
Blinding (masking)	17a	Who will be blinded after assignment to interventions (eg, trial participants, care providers, outcome assessors, data analysts), and how	3, 9
	17b	If blinded, circumstances under which unblinding is permissible, and procedure for revealing a participant's allocated intervention during the trial	3, 9
**Methods: Data collection, management, and analysis**			
Data collection methods	18a	Plans for assessment and collection of outcome, baseline, and other trial data, including any related processes to promote data quality (eg, duplicate measurements, training of assessors) and a description of study instruments (eg, questionnaires, laboratory tests) along with their reliability and validity, if known. Reference to where data collection forms can be found, if not in the protocol	6 - 8
	18b	Plans to promote participant retention and complete follow-up, including list of any outcome data to be collected for participants who discontinue or deviate from intervention protocols	6 - 12
Data management	19	Plans for data entry, coding, security, and storage, including any related processes to promote data quality (eg, double data entry; range checks for data values). Reference to where details of data management procedures can be found, if not in the protocol	6 - 12
Statistical methods	20a	Statistical methods for analysing primary and secondary outcomes. Reference to where other details of the statistical analysis plan can be found, if not in the protocol	13 - 17
	20b	Methods for any additional analyses (eg, subgroup and adjusted analyses)	13 - 17
	20c	Definition of analysis population relating to protocol non-adherence (eg, as randomised analysis), and any statistical methods to handle missing data (eg, multiple imputation)	13 - 17
**Methods: Monitoring**			
Data monitoring	21a	Composition of data monitoring committee (DMC); summary of its role and reporting structure; statement of whether it is independent from the sponsor and competing interests; and reference to where further details about its charter can be found, if not in the protocol. Alternatively, an explanation of why a DMC is not needed	12

	21b	Description of any interim analyses and stopping guidelines, including who will have access to these interim results and make the final decision to terminate the trial	12
Harms	22	Plans for collecting, assessing, reporting, and managing solicited and spontaneously reported adverse events and other unintended effects of trial interventions or trial conduct	12
Auditing	23	Frequency and procedures for auditing trial conduct, if any, and whether the process will be independent from investigators and the sponsor	12
**Ethics and dissemination**			
Research ethics approval	24	Plans for seeking research ethics committee/institutional review board (REC/IRB) approval	17-18
Protocol amendments	25	Plans for communicating important protocol modifications (eg, changes to eligibility criteria, outcomes, analyses) to relevant parties (eg, investigators, REC/IRBs, trial participants, trial registries, journals, regulators)	18
Consent or assent	26a	Who will obtain informed consent or assent from potential trial participants or authorised surrogates, and how (see Item 32)	9
	26b	Additional consent provisions for collection and use of participant data and biological specimens in ancillary studies, if applicable	16-17
Confidentiality	27	How personal information about potential and enrolled participants will be collected, shared, and maintained in order to protect confidentiality before, during, and after the trial	6-7, 12
Declaration of interests	28	Financial and other competing interests for principal investigators for the overall trial and each study site	21
Access to data	29	Statement of who will have access to the final trial dataset, and disclosure of contractual agreements that limit such access for investigators	-
Ancillary and post-trial care	30	Provisions, if any, for ancillary and post-trial care, and for compensation to those who suffer harm from trial participation	10
Dissemination policy	31a	Plans for investigators and sponsor to communicate trial results to participants, healthcare professionals, the public, and other relevant groups (eg, via publication, reporting in results databases, or other data sharing arrangements), including any publication restrictions	18
	31b	Authorship eligibility guidelines and any intended use of professional writers	-
	31c	Plans, if any, for granting public access to the full protocol, participant-level dataset, and statistical code	-

For peer review only - http://bmjopen.bmj.com/site/about/guidelines.xhtml

Appendices

Informed consent materials	32	Model consent form and other related documentation given to participants and authorised surrogates	-
Biological specimens	33	Plans for collection, laboratory evaluation, and storage of biological specimens for genetic or molecular analysis in the current trial and for future use in ancillary studies, if applicable	16-17

*It is strongly recommended that this checklist be read in conjunction with the SPIRIT 2013 Explanation & Elaboration for important clarification on the items. Amendments to the protocol should be tracked and dated. The SPIRIT checklist is copyrighted by the SPIRIT Group under the Creative Commons "Attribution-NonCommercial-NoDerivs 3.0 Unported" license.

For peer review only

BMJ Open

SALpingectomy for STERilisation (SALSTER); Study protocol for a Swedish multicentre register-based randomised controlled trial.

Journal:	*BMJ Open*
Manuscript ID	bmjopen-2022-071246.R1
Article Type:	Protocol
Date Submitted by the Author:	11-Jul-2023
Complete List of Authors:	Magarakis, Leonidas; University of Gothenburg, Department of Obstetrics and Gynecology<br>Idahl, Annika ; Umea Universitet,<br>Sundfeldt, Karin; University of Gothenburg, Department of Obstetrics and Gynecology; Sahlgrenska University Hospital, Department of Obstetrics and Gynecology<br>Liv, Per; Umeå University, Department of Public Health and Clinical Medicine, Section of Sustainable Health<br>Pålsson, Mathias; University of Gothenburg, Department of Obstetrics and Gynecology<br>Strandell, Annika; University of Gothenburg Institute of Clinical Sciences
**Primary Subject Heading**:	Obstetrics and gynaecology
Secondary Subject Heading:	Obstetrics and gynaecology
Keywords:	Minimally invasive surgery < GYNAECOLOGY, Gynaecological oncology < GYNAECOLOGY, GYNAECOLOGY

SCHOLARONE™
Manuscripts

SALpingectomy for STERilisation (SALSTER); Study protocol for a Swedish multicentre register-based randomised controlled trial.

Leonidas Magarakis[a*], Annika Idahl[b], Karin Sundfeldt[a,c], Per Liv[d], Mathias Pålsson[a] and Annika Strandell[a,c]

[a] Department of Obstetrics and Gynecology, Institute of Clinical Sciences, Sahlgrenska Academy, University of Gothenburg, Sweden.

[b] Department of Clinical Sciences, Obstetrics and Gynecology, Umeå University, Umeå, Sweden.

[c] Department of Obstetrics and Gynecology, Sahlgrenska University Hospital, Gothenburg, Sweden.

[d] Department of Public Health and Clinical Medicine, Umeå University, Umeå, Sweden.

*Corresponding author:

Leonidas Magarakis, Department of Obstetrics and Gynecology, Skåne University Hospital, Jan Waldenströms gata 47, SUS, Malmö, Sweden.

email: leonidas.magarakis@gu.se, tel: +4640332541

For peer review only

Word count abstract: 243 (Limit 250)

Word count manus: 3982 (Limit 4000)

Keywords: Sterilisation, Salpingectomy, Tubal ligation, Tubal occlusion, Randomised controlled trial, Ovarian cancer

Abstract

Introduction

Salpingectomy is currently suggested as an alternative to tubal ligation for sterilisation. Precursor lesions of ovarian carcinoma can be found in the Fallopian tubes; thus, salpingectomy could possibly reduce the incidence. Most of the existing trials on safety are small, on caesarean section and report on surrogate ovarian function measures. Randomised trials in laparoscopy are lacking. Well-designed trials are needed to evaluate safety of laparoscopic opportunistic salpingectomy.

Methods and analysis

In SALSTER, a national register-based randomised controlled non-inferiority trial, 968 women <50 years, wishing laparoscopic sterilisation will be randomised to either salpingectomy or tubal ligation. The Swedish National Quality Register of Gynecological Surgery (GynOp) will be used for inclusion, randomisation, and follow-up. Primary outcomes are *any complication* up to eight weeks postoperatively, and *age at menopause*. Both outcomes are measured with questionnaires, complications are also assessed by a gynaecologist. In a nested trial, ovarian function will be evaluated comparing the mean difference of anti-Müllerian hormone, assessed preoperatively and one year after surgery.

Ethics and dissemination

Performing salpingectomy for sterilisation has become increasingly common, despite the unclear risk-benefit balance. SALSTER studies the safety of salpingectomy compared with tubal ligation. Regardless of the result, SALSTER will provide gynaecologists with high quality evidence to inform women to decide on salpingectomy or not. The central ethical

For peer review only - http://bmjopen.bmj.com/site/about/guidelines.xhtml

review board of Gothenburg, Sweden (Dnr. 316-18) approved the trial in 2018. Results will be presented at scientific congresses and published in peer reviewed scientific journals. The results will be communicated through professional organisations and research networks.

Registration details

ClinicalTrials.gov, NCT03860805. Registered March 4th, 2019. Study protocol last updated July 7th, 2023

Strengths and limitations of the study

- The register-based randomised controlled trial combines the advantages of two study designs: the randomised trial with unbiased allocation to minimise confounding and the observational register study with an automated and cost-efficient follow-up.
- Using the GynOp register as a platform allows all trial components (identification of eligible patients, communication regarding study information and giving informed consent, randomisation, and follow-up questionnaires) to be conducted within the register.
- The use of the Swedish personal identification number allows cross-linking of the study cohort with multiple registers for the long-term follow-up.
- The multicentre design enhances the generalisability of the results.
- The nature of the trial makes blinding of the patients very difficult and impossible for the surgeons.

INTRODUCTION

For peer review only

The use of salpingectomy as a sterilisation procedure is increasing, due to the theory of high-grade serous ovarian carcinoma (HGSC) originating from the Fallopian tube. Epithelial ovarian cancer (EOC) is a group of heterogeneous malignancies regarding origin, molecular biology, morphology, gene expression, and clinical behaviour. Precancerous lesions, serous tubal intraepithelial carcinomas (STIC), detected in the tubal epithelium are suggested to be the origin of EOC, particularly HGSC. Dysplastic cells may shed from STIC lesions and implant on the ovaries and/or peritoneum and develop into HGSC.(1) Opportunistic salpingectomy to remove the potential site of origin as a preventive measure is therefore suggested for women who wish permanent sterilisation.(2,3)

Tubal ligation is by itself associated with some protection against EOC.(4) Fallopian tubes may act as a conduit of either malignant or normal cells from the endometrial cavity to the ovaries. These cells may give rise to endometrioid and clear-cell carcinomas directly or indirectly by malignant transformation of benign conditions such as endometriosis.(5) Possibly, salpingectomy could add to the protective effect of tubal ligation by removing the fimbriated end of the Fallopian tubes where STIC lesions may develop.(4,6,7)

Several gynaecological societies recommend physicians to inform women planned to undergo sterilisation, that bilateral salpingectomy instead of tubal ligation, is an option.(2,3) This recommendation is based on observational studies showing that *indicated* salpingectomy compared with no surgery, is associated with a decreased EOC incidence.(4,6,7) The effect size of *opportunistic* salpingectomy compared with tubal ligation is unknown.

There are safety concerns, since salpingectomy increases surgical trauma compared with tubal ligation. This may increase perioperative complications and may also affect blood and

For peer review only - http://bmjopen.bmj.com/site/about/guidelines.xhtml

nerve supply to the ovaries, impairing ovarian function, and possibly, in the long term, cause an earlier menopause.(8) Systematic reviews comparing salpingectomy with tubal ligation for safety outcomes such as reoperation, intraoperative complications, blood loss, wound infections etc, have identified studies with various limitations.(9) All published randomised controlled trials (RCTs) are small and conducted at caesarean section. They report on surrogate measures of endocrine function and demonstrate no difference in the short term.(10-12) Many of the published cohort studies are small and underpowered to study complications. Sterilisation is more commonly performed by laparoscopy, especially after the hysteroscopic salpingeal occluding technique with permanent implants was withdrawn from the market due to adverse effects.(13) No trial has reported on the outcome EOC. A large retrospective cohort study detected no difference in time to menopausal symptoms when comparing women who had undergone salpingectomy or tubal ligation. However, the follow-up period was insufficiently short to analyse menopausal symptoms.(14) Well-designed randomised trials of laparoscopic sterilisation procedures are needed to compare salpingectomy with tubal ligation regarding both surgical outcomes and clinical endpoints of ovarian function.

This register-based randomised trial will study the safety of laparoscopic salpingectomy for sterilisation compared with tubal ligation. The specific aim is to analyse if the risk of complications and hormonal side effects do not increase beyond pre-defined non-inferiority margins after salpingectomy compared with tubal ligation.

METHODS AND ANALYSIS

General study design

For peer review only

SALSTER, a national register-based, randomised controlled trial (R-RCT) will compare two laparoscopic procedures for sterilisation: salpingectomy and tubal ligation for safety aspects, in women without known hereditary risk for EOC.

In the primary analyses, SALSTER will test the hypotheses that salpingectomy compared with tubal ligation for laparoscopic sterilisation,

- does not increase the risk for complications perioperatively and up to eight weeks postoperatively.
- does not cause earlier menopause, assessed as age at onset of natural menopause.

The GynOp register

The SALSTER trial is conducted within the Swedish National Quality Register of Gynecological Surgery (GynOp).(15) GynOp is used by all gynaecological departments in Sweden. Inclusion and participation in national quality registers in Sweden is regulated by law (16); patients are informed of their inclusion in the register, with an "opt-out" clause which, if activated, enables the patient to have all his or her data removed from the register. The GynOp database is approved for use by health-care systems under the supervision of the Swedish Data Protection Authority. All information is stored on secured servers at Region Västerbotten. Background health data, information on surgical procedures, diagnoses, complications at eight weeks and one year postoperatively are routinely recorded in GynOp. Women planned for gynaecologic surgery receive a personal password that allows them to logon to GynOp to answer pre-operative and follow-up questionnaires. Data input in GynOp is mainly web-based, but printouts of questionnaires can be used if needed. The data collection forms and questionnaires are available from www.gynop.org on request.

For peer review only - http://bmjopen.bmj.com/site/about/guidelines.xhtml

All gynaecological departments reporting data to the register received information about the trial and were automatically included unless a department actively declined participation. A list of gynaecological departments participating in the study can be provided by the GynOp office in Umeå on demand. Both regional and academic gynaecological departments are participating in the study. The Swedish network for National Clinical Studies in Obstetrics and Gynecology (SNAKS) is actively involved and improves collaboration between health care providers engaged in the trial.(17)

A specific SALSTER application has been added to GynOp to complement existing routines. This module includes screening of eligibility, presentation of study information and opportunity to give informed consent on-line, as well as randomisation and trial-specific questionnaires pre-operatively and for follow-up.

Preoperatively, basic baseline demographic variables are registered routinely. Added to these variables are questions on menstruation pattern, age at menarche, duration of breast feeding, previous and present use of hormonal contraceptives and previous Chlamydia infection or salpingitis to assess factors suggested to effect risk for EOC. Furthermore, the Menopause Rating Scale (MRS) (18) was added.

MRS is a validated questionnaire available in several languages, including Swedish. It has 11 questions on sweating, heart discomfort, sleep problems, depressive mood, irritability, anxiety, physical and mental exhaustion, sexual problems, bladder problems, vaginal dryness, and joint and muscular function, to which patients respond in a five-grade Likert scale.(19)

Perioperative variables in GynOp are type of anaesthesia, any pathological finding in the abdomen, procedure(s) performed, complications, use of antibiotics, operative time, route of specimen removal from the abdomen, blood loss, type of suturing and codes for surgery.

For peer review only - http://bmjopen.bmj.com/site/about/guidelines.xhtml

SALSTER-specific questions concern total number and size of trocars used, method for tubal ligation, type of devices applied for salpingectomy and tubal ligation, specific questions on method of specimen extraction and need to suture the muscle fasciae following specimen evacuation.

GynOp automatically sends questionnaires to the patients electronically at eight weeks and one year postoperatively, to assess use of analgesics, bleeding, low urinary tract symptoms, sick leave, time to daily activities, satisfaction after surgery, complications and their treatment. If no answer is received, two digital reminders are sent automatically, and thereafter by ordinary mail. Patient-reported complications are assessed and documented by a gynaecologist. Any complication is registered according to the Clavien-Dindo classification.(20) No amendments have been made to the eight-weeks questionnaire.

The one-year questionnaire holds questions relating to pain experience, oestrogen treatment, symptoms from vagina, bladder and rectum, sexual intercourse last three months, coitus pain, result and satisfaction after surgery, complications, treatment of complications, hospital care, and sick leave. The questionnaire has been supplemented with trial-specific questions on oestrogen and/or progesterone hormonal treatments and their indication, MRS, menstruation pattern, unintended pregnancies and their outcomes, and smoking habits.

Routinely there is no further follow up from GynOp. For trial participants questionnaires are sent every other year until the age of 55. Questions relate to the use of menopausal hormone therapy (MHT) or oestrogen and/or progesterone hormonal treatments and their indication, MRS, bleeding pattern, smoking habits, and unintended pregnancies and their outcomes.

Eligibility

For peer review only - http://bmjopen.bmj.com/site/about/guidelines.xhtml

All patients planned for laparoscopic sterilisation are automatically screened for eligibility in the trial by the GynOp software. Patients with a known hereditary susceptibility for EOC such as BRCA mutations are not considered for tubal ligation and thus not for inclusion in SALSTER. Potential trial participants can read on-line the SALSTER information and answer the specific study questions. Paper printouts are also available in which case a medical administrator registers the information in GynOp by using a login with a two-factor authentication system. Patients may also be informed about the trial at an out-patient clinic visit when the decision on sterilisation is taken. Informed consent (Appendix 1) can be given, usually on-line within GynOp or by signing a paper document at any time point before randomisation. The consent is kept safe according to established research routines. Inclusion and exclusion criteria are summarised in Table 1.

Table 1. Eligibility criteria for women participating in SALSTER

**Inclusion criteria**	**Exclusion criteria**
Scheduled for laparoscopic sterilisation.<br>Willing to be randomised.	Women older than 49 years.<br>Not able to understand oral and written study information.<br>Previously treated for malignancy with either chemo-, radio- or hormonal therapy which may negatively affect ovarian function.

Randomisation and blinding

The randomisation module in GynOp randomly allocates women in proportion 1:1 to either salpingectomy or tubal ligation using permuted blocks with random sizes of either two or four while stratified for centre. Timing of randomisation is as close as possible to the time of surgery. The randomisation is performed on-line by the examining/operating gynaecologist or assistant with an immediate allocation response.

The nature of the trial makes blinding of patients very difficult and impossible for surgeons. Our intention is to avoid revealing information about which type of surgery was performed and we ask trial participants not to read their on-line medical records. However, the right to

For peer review only - http://bmjopen.bmj.com/site/about/guidelines.xhtml

read medical records is regulated by law. Blinding of patients is further aggravated as a detailed preoperative information is given including the number of scars associated with each procedure. In general, tubal ligation requires only one accessory port whereas salpingectomy requires at least two. Hence, blinding is not guaranteed.

Interventions

Both interventions are planned as laparoscopic procedures. If the allocated procedure cannot be executed because of either unexpected pathology or high risk for serious intraoperative complications, the surgical procedure that was eventually performed will be registered in GynOp, but the individual still contributes with follow-up data. The same applies if extra surgical procedures are needed or in case of conversion to laparotomy where all surgical interventions are registered.

Follow-up

Hospital staff routinely register data in GynOp at the end of every surgical procedure and at discharge. In case of a complication the surgeon registers the event. Responsible surgeon assesses the eight-weeks and one-year questionnaires and in suspicion of a complication or unsatisfactory surgical results, a consultation is arranged. Any adverse effect is registered in GynOp. If there is no response reminders, a member of the steering group contacts the department. In every department, a responsible physician will check responses and completeness of questionnaires at different time points. In case of an adverse event, any need for medical treatment to trial participants is covered by the Swedish health care system according to the Swedish law.

Outcomes

For peer review only - http://bmjopen.bmj.com/site/about/guidelines.xhtml

The trial has two primary outcomes, one in the short- and one in the long-term. Secondary outcomes are registered in the short-, intermediate- and long-term (Table 2).

Table 2. Outcomes in SALSTER

**Time interval**	**Primary outcomes**	**Secondary outcomes**
Short term<br>*(up to 8 weeks)*	Any complication	Severe complications<br>Operative time<br>Perioperative blood loss.<br>Length of hospital stay
Intermediate term<br>*(one year after surgery)*		Complications according to Clavien-Dindo<br>Complications according to the existing questions on complications in GynOp
Intermediate and long term		Subsequent surgery on uterus, salpinges and/or ovaries<br>Pregnancy rate
Long term<br>*(more than one year and up to 30 years after surgery)*	Age at onset of natural menopause	Age at the start of the perimenopausal state<br>Length of the perimenopausal state<br>Change in menopausal symptom score<br>Use of menopausal hormone therapy at any time during follow-up<br>Secondary expressions of oestrogen deficiency<br>Epithelial ovarian cancer

Any complication up to eight weeks post-operatively, is retrieved directly from the GynOp database. The outcome includes any complication occurring per-operatively, diagnosed at postoperative emergency visits, or noted by the patient and assessed by the physician in the eight-weeks questionnaire. The complication is further categorised as mild or severe, by organ damaged, and is graded according to the Clavien-Dindo classification. These categorised variables will be analysed as secondary outcomes.

Age at onset of natural menopause, defined as twelve months of amenorrhea, is assessed by analysing reported bleeding pattern in the study-specific questionaries sent every other year. Women with MHT prescription, oestrogen and/or progesterone hormonal treatments, or a

For peer review only

subsequent hysterectomy will not be included in this primary outcome, since they do not have a natural menopause.

The secondary short-term outcomes relate to the surgery and the in-hospital care as registered in GynOp. Secondary intermediate-term outcomes are retrieved from GynOp and other national quality and health registers. Secondary long-term outcomes such as length of and age at the start of perimenopausal state will be assessed by the trial-specific questionnaires describing bleeding pattern. Need for MHT will be assessed by every-other-year questionaries and through The Drug Prescription Register up to 30 years after surgery. Uterine and adnexal surgery that occurs after the primary surgery will be assessed through GynOp at one year and The Patient register lifelong after surgery. Unintended pregnancies and their outcomes will be registered through the trial-specific questionnaires. If outcomes on ovarian function show a difference between groups, consequences of oestrogen deficiency, i.e., fractures related to osteoporosis and cardio-vascular events will be assessed through The Patient register.

Ovarian cancer will be assessed by cross-linking SALSTER with Swedish national registers and pooled with data from the ongoing Hysterectomy and OPPortunistic Salpingectomy (HOPPSA) trial. HOPPSA is a Swedish multi-centre, register-based RCT where patients planned for hysterectomy are randomised to salpingectomy or no salpingectomy.(21) By pooling data from SALSTER and HOPPSA the effect size of opportunistic salpingectomy to reduce the incidence of epithelial ovarian cancer will be estimated. Data will be retrieved through The Swedish Cancer Register, The Swedish Quality Register for Gynaecological Cancer, The Swedish Cause of Death Register and The Swedish Population Register and at lifelong follow-up.

For peer review only - http://bmjopen.bmj.com/site/about/guidelines.xhtml

Data monitoring and data management

Each surgical procedure in GynOp automatically receives a unique identification code number. This number is used in the trial to assign individual data, thus protecting confidentiality. The number of individuals randomised in the trial is continuously monitored by the GynOp's administrators. Numbers of recruited and percentage of eligible women per participating clinic are reported every three months on the GynOp website and through the SNAKS network which enhances communication between the research group and the departments participating in the trial. Regular online meetings are being held updating departments on the progress of the trial, and information is shared on recruiting performance. An independent appointed Data Safety Monitoring Board has performed an interim analysis when 50% of the target sample size was reached, according to the original plan, and gave clearance for the study to continue recruiting patients.

Patient and public involvement

Women in reproductive age in the general population were involved at an early phase of the planning, regarding choice of outcomes and development of the written study information. A short explanation of the research question and the intended study protocol in lay language with suggested outcomes were distributed among volunteers in waiting rooms at gynaecology departments in Sweden. Open and specific questions were asked concerning the relevance of the trial, the design, the outcomes, any missing issues, or missing outcomes. Questions associated with the draft of the written study information related to readability, unnecessary or missing information. Women were also asked to rate the importance of receiving information about potential risks associated with opportunistic salpingectomy.

For peer review only

STATISTICS

Sample size calculations

Primary short-term outcome: *any complication* up to eight weeks

Complications to laparoscopic tubal ligation was registered in GynOp at a rate of 13.6% from 2010 to 2017. An increase of 3% is estimated after salpingectomy. If the non-inferiority margin is defined as +10%, the upper limit of the two-sided 95% CI (α=0.05) for the difference between the salpingectomy and the tubal ligation groups shall not be above the +10% with a probability of 80% (β=20%). To demonstrate non-inferiority, 411 women per randomisation group are needed (based on a two-sided Farrington-Manning test).[22] For protection against a 10% loss to follow-up, the target sample was determined at 914. The interim analysis revealed that 5% of randomised women interrupted their participation. For protection against this loss, the target sample size was increased to 968.

Primary long-term outcome: *age at onset of menopause*

Age at menopause on a Swedish population level was reported to be in mean 51.5 years and SD was estimated at 3.0. A decrease of one year is estimated after salpingectomy. If the non-inferiority margin is defined as two years, the upper limit of the two-sided 95% CI (α=0.05) for the difference between the salpingectomy group and the tubal ligation group shall not be above two years with a probability of 80% (β=20%). To demonstrate non-inferiority, 143 women per randomisation group are needed (two-sided non-parametric permutation test for comparison of two means). Considering exclusion of women without a natural menopause (30%), 5% of randomised women interrupting participation before the eight-weeks questionnaire, and 15% loss during the 20 years long follow-up, approximately 572 women are needed for recruitment.

For peer review only - http://bmjopen.bmj.com/site/about/guidelines.xhtml

Statistical plan

Both “intention to treat”, and “per protocol” analyses will be performed. For non-inferiority design, the “per protocol” analysis will be the primary.

Any complication will be presented as numbers along with percentages with 95% CI and the *age at onset of menopause* will be presented as mean and standard deviations, as well as with median and quartiles. The two primary analyses measure different outcomes at different time points and will be published in separate articles. As they also test two different hypotheses, we will refrain from adjusting the 5% significance level for multiplicity.

Analyses of *any complication up to eight weeks post-operatively*

Primary analysis: To account for the lack of independence introduced by the stratification of the randomisation, we will estimate the difference in the complication risk between the two randomised groups with a 95% CI using a generalised estimation equation (GEE) with logistic link function, marginalised over centre, and adjusted for age. The 95% CI of the risk difference will be estimated from the GEE-model using the delta method. The upper limit of the 95% CI shall not exceed the non-inferiority margin of 10%. As a sensitivity analysis, the unadjusted 95% CI for the difference in complications will be calculated according to Ferrington-Manning.(22) Furthermore, unadjusted risk ratio (RR) and adjusted RR with 95% CI will also be calculated in secondary analyses using a GEE Poisson model with robust standard errors.

Analyses of *age at menopause*

For peer review only

The primary analysis will be a mixed effect model with adjustment for age as fixed effect and centre as random effect, from which a two-sided 95% CI for the mean difference will be constructed. The upper limit of the 95% CI shall not exceed the non-inferiority margin of two years for non-inferiority to be established. A sensitivity analysis without adjustment will be conducted by constructing a 95% CI for the mean difference using Fisher's non-parametric permutation test.

Missing data on the primary outcomes will be replaced with multiple imputation using fully conditional specification in the main analysis. In addition, a complete case analysis will be conducted. If both analyses of the two primary outcomes demonstrate non-inferiority, a common conclusion on the safety of the intervention can be inferred. However, the long period between these analyses will entail separate conclusions on complications and age at menopause, in a temporal order.

For other unadjusted comparisons between the two randomised groups Fisher's non-parametric permutation test will be used for continuous variables, Mantel-Haenszel Chi^2-test for ordered categorical variables, Fisher's exact test for dichotomous variables and Chi^2-test for non-ordered categorical variables. For dichotomous outcomes, a two-sided 95% CI for the difference in proportions between groups will be calculated as well as risk ratios with 95% CI. For continuous outcomes, two-sided 95% CIs for the difference in means between groups will be calculated. Also, adjusted analyses will be conducted.

All results from the secondary analysis will be given with estimates, 95% CI and two-sided p-values, as well as unadjusted and adjusted RR with 95% CI. The analyses of the secondary endpoints will be mainly explanatory.

For peer review only - http://bmjopen.bmj.com/site/about/guidelines.xhtml

A detailed statical analysis plan (SAP) will be written before data retrieval and published at the trial's site at ClinicalTrials.gov. Updates and changes in the planned statistical analyses will be published there.

NESTED TRIAL OF ANTI-MÜLLERIAN HORMONE LEVELS

A biochemical measure of ovarian function is the serum level of AMH, a product of granulosa cells of the preantral and small antral follicles in the ovaries.(23) There is a theoretical rationale that salpingectomy may disturb the vascular and nervous supply to the ovary, or disrupt paracrine signalling, possibly causing impairment in ovarian function.(8) In the main trial, the primary outcome for ovarian function is based on clinical symptoms related to menopause. To strengthen the hypothesis of non-inferiority for ovarian function if salpingectomy is performed, an analysis of AMH is planned in a subset of patients.

Consecutive patients in SALSTER are asked for blood samples. Specific written and oral information is provided, and informed consent is signed. Blood samples are drawn at baseline and after one year. Seven hospitals are engaged in this nested trial. Samples are handled according to laboratory instructions, centrifugated, frozen within two days and stored in a biobank for later analysis, when the entire cohort will be analysed at the same time.

Results will be available after one year of follow-up and added manually to the GynOp dataset. Patients wishing to be informed about their AMH levels result will be contacted. AMH levels will be compared between the salpingectomy vs. tubal ligation groups and

For peer review only - http://bmjopen.bmj.com/site/about/guidelines.xhtml

presented both in absolute and relative measures. Primary endpoint is absolute change in AMH from baseline to one year after surgery.

If non-inferiority is defined as 0.2 mg/L AMH, the upper limit of the two-sided 95% CI for the difference in change between the two groups shall not exceed 0.2 (SD for change 0.45) with a probability of 80% (β=20%), and an estimation of up to 0.0 larger change (no difference in change) in the salpingectomy group, 81 patients per randomisation group is needed to show non-inferiority. Estimating a 20% loss to follow-up (a second blood sample not taken), 204 patients will be recruited in this nested trial. A two-sided 95% CI for the mean difference in absolute change in AMH will be constructed using a mixed effect model with adjustment for age as fixed effect and centre as random effect. Fisher's non-parametric permutation test will be applied for the unadjusted analysis.

ETHICS AND DISSEMINATION

Even though EOC is not the most common gynaecological cancer it carries the worst prognosis due to early spread and vague symptomatology, making diagnosis difficult at an early stage. Based on the theory that the most common and aggressive form, HGSC may arise from the epithelium of the Fallopian tubes, the practice of opportunistic salpingectomy has rapidly gained popularity. Well-designed trials have not been performed to study the safety profile of salpingectomy compared with tubal ligation regarding complications and the effect on ovarian function. SALSTER will assess if salpingectomy is as safe as tubal ligation. The withdrawal of hysteroscopic sterilisation made the trial ethically reasonable to design since the less invasive hysteroscopic procedure for sterilisation was not available anymore.(13) Regardless of the result, the trial will provide gynaecologists with high quality evidence to

For peer review only

inform women, who can decide on having their tubes removed or not. If no additional risk is found, salpingectomy can be a recommended option. If not, the risks and benefits should be considered when counselling women wishing permanent surgical sterilisation.

SALSTER does not have EOC as a primary outcome for several reasons: There is a parallel trial, HOPPSA, which has EOC as a long-term primary outcome. At inclusion, the patients in HOPPSA are older than those in SALSTER, which implies a shorter time-to-event than in SALSTER. Also, hysterectomy is a more frequent procedure than sterilisation in Sweden, implying faster recruitment to the target sample size. Thus, the HOPPSA trial is more suited to investigate and conclude on EOC as a primary outcome. Furthermore, the plan for SALSTER is to contribute data to be pooled with HOPPSA data for the evaluation of the effect of opportunistic salpingectomy on EOC. A combined SAP will be written for an IPD meta-analysis combining HOPPSA and SALSTER.

The results of this trial will be presented at national as well as international scientific congresses and several publications are planned in international scientific journals. All results will be presented on aggregated level, without any possibility to identify individuals. SNAKS will help to spread the results of this trial to its network of gynaecological departments in Sweden. Updates of results will be presented at the annual meetings of the Swedish Society of Obstetrics and Gynecology.

The SALSTER trial was approved by the central ethical review board in Gothenburg, Sweden June 18th, 2018 (Dnr. 316-18). The first patient was randomised April 4th, 2019. The trial is recruiting, and 864 women had been randomised August 31st, 2022.

Authors' contribution

For peer review only

For peer review only - http://bmjopen.bmj.com/site/about/guidelines.xhtml

AS initiated the trial, designed, and drafted the first study protocol. AI engaged in the revision and editing of the protocol. AI and MP are the primary contact persons with the GynOp register. KS contributes with ovarian tumour biology experience. LM initiated the AMH nested trial. AS, AI, KS, MP, and LM approved the study protocol. AS applied to the Swedish Ethical Review Authority. PL wrote the statistical plan. LM wrote the first draft of this manuscript which was revised by AS, AI, KS, and MP. All committed authors approved the final version of this manuscript.

Principal investigator: Annika Strandell, contact email: annika.strandell@vgregion.se

Funding statement

This work was supported by the Swedish Cancer Society (21 1408 Pj), the Lena Wäppling foundation, the Swedish state under the agreement between the Swedish government and the county councils, the ALF-agreement (ALFGBG-965130 and ALF RegVB-969584), Umeå University and Center for clinical research, county of Värmland grant number (LIVFOU-929703). The funders were not involved in study design; collection, management, analysis, and interpretation of data; writing of the report; and the decision to submit the report for publication.

Competing interest statement

None of the other authors have any conflicts of interest.

REFERENCES

1. Prat J. New insights into ovarian cancer pathology. *Annals of oncology : official journal of the European Society for Medical Oncology* 2012;23 Suppl 10:111-7. doi: https://doi.org/10.1093/annonc/mds300

For peer review only - http://bmjopen.bmj.com/site/about/guidelines.xhtml

2. Salvador S, Scott S, Francis JA, et al. No. 344-Opportunistic Salpingectomy and Other Methods of Risk Reduction for Ovarian/Fallopian Tube/Peritoneal Cancer in the General Population. *Journal of obstetrics and gynaecology Canada : JOGC* 2017;39(6) doi: https://10.1016/j.jogc.2016.12.005
3. ACOG Committee Opinion No. 774: Opportunistic Salpingectomy as a Strategy for Epithelial Ovarian Cancer Prevention. *Obstetrics and gynecology* 2019;133(4) doi: https://10.1097/AOG.0000000000003164
4. Madsen C, Baandrup L, Dehlendorff C, et al. Tubal ligation and salpingectomy and the risk of epithelial ovarian cancer and borderline ovarian tumors: a nationwide case-control study. *Acta obstetricia et gynecologica Scandinavica* 2015;94(1):86-94. doi: https://doi.org/10.1111/aogs.12516
5. Cochrane DR, Tessier-Cloutier B, Lawrence KM, et al. Clear cell and endometrioid carcinomas: are their differences attributable to distinct cells of origin? *The Journal of pathology* 2017;243(1) doi: https://10.1002/path.4934
6. Lessard-Anderson CR, Handlogten KS, Molitor RJ, et al. Effect of tubal sterilization technique on risk of serous epithelial ovarian and primary peritoneal carcinoma. *Gynecologic oncology* 2014;135(3) doi: https://doi.org/10.1016/j.ygyno.2014.10.005
7. Falconer H, Yin L, Grönberg H, et al. Ovarian cancer risk after salpingectomy: a nationwide population-based study. *Journal of the National Cancer Institute* 2015;107(2):410. doi: https://doi.org/10.1093/jnci/dju410
8. Kotlyar A, Gingold J, Shue S, et al. The Effect of Salpingectomy on Ovarian Function. *Journal of minimally invasive gynecology* 2017;24(4) doi: https://10.1016/j.jmig.2017.02.014
9. Magarakis L, Idahl A, Särnqvist C, et al. Efficacy and safety of sterilisation procedures to reduce the risk of epithelial ovarian cancer: a systematic review comparing salpingectomy with tubal ligation. *The European journal of contraception & reproductive health care : the official journal of the European Society of Contraception* 2021 doi: https://10.1080/13625187.2021.2010041
10. Ganer Herman H, Gluck O, Keidar R, et al. Ovarian reserve following cesarean section with salpingectomy vs tubal ligation: a randomized trial. *American journal of obstetrics and gynecology* 2017;217(4):472.e1-72.e6. doi: https://doi.org/10.1016/j.ajog.2017.04.028
11. Garcia C, Moskowitz OM, Chisholm CA, et al. Salpingectomy Compared With Tubal Ligation at Cesarean Delivery: A Randomized Controlled Trial. *Obstetrics and gynecology* 2018;132(1):29-34. doi: https://doi.org/10.1097/AOG.0000000000002674
12. Subramaniam A, Blanchard CT, Erickson BK, et al. Feasibility of Complete Salpingectomy Compared With Standard Postpartum Tubal Ligation at Cesarean Delivery: A Randomized Controlled Trial. *Obstetrics and gynecology* 2018;132(1):20-7. doi: https://doi.org/10.1097/AOG.0000000000002646
13. Problems Reported with Essure. Available from: https://www.fda.gov/medical-devices/essure-permanent-birth-control/problems-reported-essure [Accessed 11th November 2022].
14. Hanley GE, Kwon JS, McAlpine JN, et al. Examining indicators of early menopause following opportunistic salpingectomy: a cohort study from British Columbia, Canada. *American journal of obstetrics and gynecology* 2020;223(2):221.e21-21.e11. doi: https://doi.org/10.1016/j.ajog.2020.02.005
15. The Swedish National Quality Register of Gynecological Surgery. Available from: http://www2.gynop.se/home/ [Accessed 7th November 2022].

For peer review only - http://bmjopen.bmj.com/site/about/guidelines.xhtml

16. Swedish National Quality Registries. Available from: https://www.kvalitetsregister.se/en/kvalitetsregister/omnationellakvalitetsregister.52218.html [Accessed 7 November 2022].
17. Swedish network for national clinical studies in Obstetrics and Gynecology. Available from: https://www.snaks.se [Accessed 7th November 2022].
18. Heinemann K, Ruebig A, Potthoff P, et al. The Menopause Rating Scale (MRS) scale: a methodological review. *Health and quality of life outcomes* 2004;2 doi: https://10.1186/1477-7525-2-45
19. Menopause Rating Scale. Available from: http://www.menopause-rating-scale.info/documents/MRS_English.pdf [Accessed 7th November 2022].
20. Dindo D, Demartines N, Clavien PA. Classification of surgical complications: a new proposal with evaluation in a cohort of 6336 patients and results of a survey. *Annals of surgery* 2004;240(2) doi: https://10.1097/01.sla.0000133083.54934.ae
21. Idahl A, Darelius A, Sundfeldt K, et al. Hysterectomy and opportunistic salpingectomy (HOPPSA): study protocol for a register-based randomized controlled trial. *Trials* 2019;20(1) doi: https://10.1186/s13063-018-3083-8
22. Farrington CP, Manning G. Test statistics and sample size formulae for comparative binomial trials with null hypothesis of non-zero risk difference or non-unity relative risk. *Statistics in medicine* 1990;9(12) doi: https://10.1002/sim.4780091208
23. Vigier B, Picard JY, Tran D, et al. Production of anti-Müllerian hormone: another homology between Sertoli and granulosa cells. *Endocrinology* 1984;114(4) doi: https://10.1210/endo-114-4-1315

For peer review only

SALPINGECTOMY AT STERILIZATION

SALSTER

Consent to take part in the SALSTER study involves

- confirming that you have received information about the study and have been given the opportunity to ask questions

- that you agree to participate in the study and that your personal data will be processed as described in the information

- that you are aware that your participation is completely voluntary and that you can cancel your participation without explanation, and without affecting your care and treatment in any future contacts with the health care system

I agree to participate in the SALSTER study

☐ Yes, I do

☐ No

☐ Maybe, but I would like more information

I agree to answer survey questions

☐ Yes

☐ No

2018-09-07

SNAKS

Swedish network for national clinical studies in Ob/Gyn

For peer review only - http://bmjopen.bmj.com/site/about/guidelines.xhtml

This is the SALSTER Consent form as it appears in a print-out. It has been translated from Swedish with www.DeepL.com/Translator

Women log on to GynOp where they read this text and give consent on-line under the protection of a secured password.

For peer review only

SPIRIT schedule of enrolment, interventions, and assessments in the SALSTER trial according to the SPIRIT guidelines

			**STUDY PERIOD**					
	**Enrolment**	**Allocation**	**Post-allocation**					
**TIMEPOINT****	***0-30 days before allocation***	**0**	***Peri-operative***	***At discharge***	***8 weeks***	***1 year***	***Every other year up to 55 years of age***	***20-30 years***
**ENROLMENT:**								
**Eligibility screen**	X							
**Informed consent**	X							
Health questionnaires	X							
Factors that may affect the risk for EOC	X							
MRS	X							
**Allocation**		X						
**INTERVENTIONS:**								
Bilateral salpingectomy			X					
Tubal ligation			X					
**ASSESSMENTS:**								
Baseline characteristics	X	X						
Perioperative variables			X	X				
SALSTER specific operative variables			X	X				
Severe complications			X	X	X	X		
Complications			X	X	X	X		
Age at the start of perimenopausal state							X	
Length of perimenopausal state							X	
Change in menopausal symptom score						X	X	
Use of MHT						X	X	
***Subsequent* surgery on uterus, salpinges and/or ovaries**						X	X	
Pregnancy						X	X	
EOC								X
**Secondary expressions of estrogen deficiency**								X

Abbreviations: EOC= Epithelial Ovarian Cancer, MRS= Menopause rating scale, MHT= Menopausal Hormone Therapy, PBL= Perioperative Blood Loss, SALSTER= SALpingectomy for STERilisation.

For peer review only

For peer review only - http://bmjopen.bmj.com/site/about/guidelines.xhtml

STANDARD PROTOCOL ITEMS: RECOMMENDATIONS FOR INTERVENTIONAL TRIALS

SPIRIT 2013 Checklist: Recommended items to address in a clinical trial protocol and related documents*

Section/item	Item No	Description	Addressed on page number
**Administrative information**			
Title	1	Descriptive title identifying the study design, population, interventions, and, if applicable, trial acronym	1
Trial registration	2a	Trial identifier and registry name. If not yet registered, name of intended registry	3
	2b	All items from the World Health Organization Trial Registration Data Set	-
Protocol version	3	Date and version identifier	3
Funding	4	Sources and types of financial, material, and other support	20
Roles and responsibilities	5a	Names, affiliations, and roles of protocol contributors	1, 20
	5b	Name and contact information for the trial sponsor	20
	5c	Role of study sponsor and funders, if any, in study design; collection, management, analysis, and interpretation of data; writing of the report; and the decision to submit the report for publication, including whether they will have ultimate authority over any of these activities	20
	5d	Composition, roles, and responsibilities of the coordinating centre, steering committee, endpoint adjudication committee, data management team, and other individuals or groups overseeing the trial, if applicable (see Item 21a for data monitoring committee)	-
**Introduction**			
Background and rationale	6a	Description of research question and justification for undertaking the trial, including summary of relevant studies (published and unpublished) examining benefits and harms for each intervention	3 - 5
	6b	Explanation for choice of comparators	3 - 5
Objectives	7	Specific objectives or hypotheses	6
Trial design	8	Description of trial design including type of trial (eg, parallel group, crossover, factorial, single group), allocation ratio, and framework (eg, superiority, equivalence, noninferiority, exploratory)	6

For peer review only - http://bmjopen.bmj.com/site/about/guidelines.xhtml

Methods: Participants, interventions, and outcomes

Study setting	9	Description of study settings (eg, community clinic, academic hospital) and list of countries where data will be collected. Reference to where list of study sites can be obtained	6
Eligibility criteria	10	Inclusion and exclusion criteria for participants. If applicable, eligibility criteria for study centres and individuals who will perform the interventions (eg, surgeons, psychotherapists)	9, Table 1
Interventions	11a	Interventions for each group with sufficient detail to allow replication, including how and when they will be administered	10
	11b	Criteria for discontinuing or modifying allocated interventions for a given trial participant (eg, drug dose change in response to harms, participant request, or improving/worsening disease)	10
	11c	Strategies to improve adherence to intervention protocols, and any procedures for monitoring adherence (eg, drug tablet return, laboratory tests)	6 - 8
	11d	Relevant concomitant care and interventions that are permitted or prohibited during the trial	9 - 10
Outcomes	12	Primary, secondary, and other outcomes, including the specific measurement variable (eg, systolic blood pressure), analysis metric (eg, change from baseline, final value, time to event), method of aggregation (eg, median, proportion), and time point for each outcome. Explanation of the clinical relevance of chosen efficacy and harm outcomes is strongly recommended	11 - 12, Table 2
Participant timeline	13	Time schedule of enrolment, interventions (including any run-ins and washouts), assessments, and visits for participants. A schematic diagram is highly recommended (see Figure)	10 - 11, Figure 1
Sample size	14	Estimated number of participants needed to achieve study objectives and how it was determined, including clinical and statistical assumptions supporting any sample size calculations	14-17
Recruitment	15	Strategies for achieving adequate participant enrolment to reach target sample size	6-9

Methods: Assignment of interventions (for controlled trials)

Allocation:

Sequence generation	16a	Method of generating the allocation sequence (eg, computer-generated random numbers), and list of any factors for stratification. To reduce predictability of a random sequence, details of any planned restriction (eg, blocking) should be provided in a separate document that is unavailable to those who enrol participants or assign interventions	9

For peer review only - http://bmjopen.bmj.com/site/about/guidelines.xhtml

Allocation concealment mechanism	16b	Mechanism of implementing the allocation sequence (eg, central telephone; sequentially numbered, opaque, sealed envelopes), describing any steps to conceal the sequence until interventions are assigned	9
Implementation	16c	Who will generate the allocation sequence, who will enrol participants, and who will assign participants to interventions	9
Blinding (masking)	17a	Who will be blinded after assignment to interventions (eg, trial participants, care providers, outcome assessors, data analysts), and how	3, 9 - 10
	17b	If blinded, circumstances under which unblinding is permissible, and procedure for revealing a participant's allocated intervention during the trial	3, 9 - 10
**Methods: Data collection, management, and analysis**			
Data collection methods	18a	Plans for assessment and collection of outcome, baseline, and other trial data, including any related processes to promote data quality (eg, duplicate measurements, training of assessors) and a description of study instruments (eg, questionnaires, laboratory tests) along with their reliability and validity, if known. Reference to where data collection forms can be found, if not in the protocol	6 - 8
	18b	Plans to promote participant retention and complete follow-up, including list of any outcome data to be collected for participants who discontinue or deviate from intervention protocols	6 - 12
Data management	19	Plans for data entry, coding, security, and storage, including any related processes to promote data quality (eg, double data entry; range checks for data values). Reference to where details of data management procedures can be found, if not in the protocol	6 - 12
Statistical methods	20a	Statistical methods for analysing primary and secondary outcomes. Reference to where other details of the statistical analysis plan can be found, if not in the protocol	14 – 18
	20b	Methods for any additional analyses (eg, subgroup and adjusted analyses)	14 – 18
	20c	Definition of analysis population relating to protocol non-adherence (eg, as randomised analysis), and any statistical methods to handle missing data (eg, multiple imputation)	14 - 18
**Methods: Monitoring**			
Data monitoring	21a	Composition of data monitoring committee (DMC); summary of its role and reporting structure; statement of whether it is independent from the sponsor and competing interests; and reference to where further details about its charter can be found, if not in the protocol. Alternatively, an explanation of why a DMC is not needed	13

	21b	Description of any interim analyses and stopping guidelines, including who will have access to these interim results and make the final decision to terminate the trial	13
Harms	22	Plans for collecting, assessing, reporting, and managing solicited and spontaneously reported adverse events and other unintended effects of trial interventions or trial conduct	13
Auditing	23	Frequency and procedures for auditing trial conduct, if any, and whether the process will be independent from investigators and the sponsor	13
**Ethics and dissemination**			
Research ethics approval	24	Plans for seeking research ethics committee/institutional review board (REC/IRB) approval	18-19
Protocol amendments	25	Plans for communicating important protocol modifications (eg, changes to eligibility criteria, outcomes, analyses) to relevant parties (eg, investigators, REC/IRBs, trial participants, trial registries, journals, regulators)	19
Consent or assent	26a	Who will obtain informed consent or assent from potential trial participants or authorised surrogates, and how (see Item 32)	9
	26b	Additional consent provisions for collection and use of participant data and biological specimens in ancillary studies, if applicable	17-18
Confidentiality	27	How personal information about potential and enrolled participants will be collected, shared, and maintained in order to protect confidentiality before, during, and after the trial	6-7, 12 - 13
Declaration of interests	28	Financial and other competing interests for principal investigators for the overall trial and each study site	20
Access to data	29	Statement of who will have access to the final trial dataset, and disclosure of contractual agreements that limit such access for investigators	-
Ancillary and post-trial care	30	Provisions, if any, for ancillary and post-trial care, and for compensation to those who suffer harm from trial participation	10
Dissemination policy	31a	Plans for investigators and sponsor to communicate trial results to participants, healthcare professionals, the public, and other relevant groups (eg, via publication, reporting in results databases, or other data sharing arrangements), including any publication restrictions	18 - 19
	31b	Authorship eligibility guidelines and any intended use of professional writers	-
	31c	Plans, if any, for granting public access to the full protocol, participant-level dataset, and statistical code	-

For peer review only - http://bmjopen.bmj.com/site/about/guidelines.xhtml

Appendices

Informed consent materials	32	Model consent form and other related documentation given to participants and authorised surrogates	Appendix 1 (Consent form)
Biological specimens	33	Plans for collection, laboratory evaluation, and storage of biological specimens for genetic or molecular analysis in the current trial and for future use in ancillary studies, if applicable	17 - 18

*It is strongly recommended that this checklist be read in conjunction with the SPIRIT 2013 Explanation & Elaboration for important clarification on the items. Amendments to the protocol should be tracked and dated. The SPIRIT checklist is copyrighted by the SPIRIT Group under the Creative Commons "Attribution-NonCommercial-NoDerivs 3.0 Unported" license.

For peer review only
